# Sex-Related Overactivation of NLRP3 Inflammasome Increases Lethality of the Male COVID-19 Patients

**DOI:** 10.3389/fmolb.2021.671363

**Published:** 2021-06-04

**Authors:** Hongliang Zhang, Yujie Tang, Jinhui Tao

**Affiliations:** Department of Rheumatology and Immunology, The First Affiliated Hospital of USTC, Division of Life Sciences and Medicine, University of Science and Technology of China, Hefei, China

**Keywords:** SARS-CoV-2, COVID-19, NLRP3 inflammasome, sex, cytokine storm, P2X7R

## Abstract

The COVID-19 pandemic, caused by SARS-CoV-2 infection, remains a dramatic threat to human life and economic well-being worldwide. Significant heterogeneity in the severity of disease was observed for patients infected with SARS-CoV-2 ranging from asymptomatic to severe cases. Moreover, male patients had a higher probability of suffering from high mortality and severe symptoms linked to cytokine storm and excessive inflammation. The NLRP3 inflammasome is presumably critical to this process. Sex differences may directly affect the activation of NLRP3 inflammasome, impacting the severity of observed COVID-19 symptoms. To elucidate the potential mechanisms underlying sex based differences in NLRP3 activation during SARS-CoV-2 infection, this review summarizes the reported mechanisms and identifies potential therapeutic targets.

## Introduction

Coronaviridae are plus-sense single strand RNA viruses subdivided into Coronavirinae and Torovirinae. Some members of the Coronavirinae including severe acute respiratory syndrome coronavirus (SARS-CoV) and Middle East respiratory syndrome coronavirus (MERS-CoV) have drawn much attention in the past decades due to their threat to human life. At present, severe acute respiratory syndrome coronavirus 2 (SARS-CoV-2) and the resulting coronavirus disease 2019 (COVID-19) is still impacting human life around the world. Significant sex and age heterogeneity is observed between asymptomatic COVID-19 patients, compared to those with mild or severe disease. Studies have repeatedly shown that severe and fatal COVID-19 cases are most prevalent among the elderly compared to young and middle-aged patients. Most young and middle-aged patients with severe illness are male (Chen et al., 2020; Gebhard et al., 2020; Klein et al., 2020; Li et al., 2020; Meng et al., 2020). Since severe cases result in higher mortality, preventing the transition from mild to severe disease could significantly reduce mortality. In particular, identifying mechanisms linking young male COVID-19 patients to severe disease would be highly relevant.

A sudden increase of inflammation is typical for the transition of COVID-19 from mild to severe or critical disease brought about by a cytokine storm (Caricchio et al., 2021). Plasma levels of IL-1β, -7, -8, -9, -10, FGF, G-CSF, GM-CSF, IFN-γ, IP-10, MCP-1, MIP-1A, MIP1-B, PDGF, TNF-α and VEGF are significantly elevated in severe patients, and the levels are related to the prognosis of patients (Huang C. et al., 2020). Preventing excessive cytokine production could thus help prevent progression from mild to severe disease.

IL-1β and its downstream effectors IL-6 and TNF-α are linked to the exacerbation of COVID-19. Clinically, targeting IL-1β, IL-6, and TNF-α have gained some success in severe COVID-19 patients (Xu X. et al., 2020; Dimopoulos et al., 2020; Rizk et al., 2020). These cytokines are produced in the earlier phase of infection by the innate immune system and are largely responsible for later induction of excessive inflammation. Preventing the secretion of these cytokines may thus help to prevent cytokine storm.

The NLRP3 inflammasome is a multiprotein complex consisting of NLRP3, ASC and procaspase-1. It recognizes both damage-associated molecular patterns (DAMPs) and pathogen-associated molecular patterns (PAMPs). After activation of the NLRP3 inflammasome, procaspase-1 is cleaved to active caspase-1, which in turn promotes the maturation and secretion of pro-inflammatory cytokines IL-1β and IL-18, triggering an inflammatory response (Schroder and Tschopp, 2010; Yang et al., 2019). NLRP3 inflammasome overactivation produces excessive IL-1β and downstream cytokines such as IL-6 and TNF-α, are also observed in acute respiratory distress syndrome (ARDS), ventilator induced lung injury (VILI), and disseminated intravascular coagulation (DIC). In addition, inflammatory damage of the heart, kidney, digestive and nervous systems result in COVID-19 patients (de Rivero Vaccari et al., 2020). Thus NLRP3 inflammasome overactivation may be responsible for SARS-CoV-2 induced cytokine storm in COVID-19 patients. Physiological and biochemical characteristics different between males and females may activate the NLRP3 inflammasome to varying degrees resulting in different inflammatory symptoms and prognosis. To further analyze the mechanisms underlying the cytokine storm after SARS-CoV-2 infection and the heterogeneity observed between males and females, this review summarizes the reported mechanisms of sex impact on NLRP3 inflammasome activation and proposes the potential therapeutic targets for COVID-19 treatment.

## Role of NLRP3 Inflammasome Activation in Patients With COVID-19

Studies have repeatedly shown that coronavirus infection can trigger the activation of NLRP3 inflammasome through different ways. Notably, the coronavirus genome-encoded proteins contribute significantly to this activation. For example, SARS-CoV genome encodes a variety of proteins (Kim et al., 2020), including viroporin that forms ion channels in the membrane and promotes an ionic flux to activate NLRP3 inflammasome. ORF8a, ORF3a and E proteins are all viroporins (Xu et al., 2020a). E protein forms ion channels on the ERGIC/Golgi membrane and mediates the influx of calcium ions to activate NLRP3 inflammasome (Nieto-Torres et al., 2015). ORF3 induces NLRP3 inflammasome activation not only in an ion channel-dependent manner (Chen et al., 2019), but also via TNF receptor-associated factor 3 (TRAF3)-mediated ASC ubiquitination (Siu et al., 2019). Other proteins, such as SUD and ORF8b, can also activate NLRP3 inflammasome (Shi et al., 2019; Chang et al., 2020). Since SARS-CoV-2 shares 79.6% sequence identity with SARS-CoV (Zhou et al., 2020), it is reasonable to infer that the molecular mechanisms of NLRP3 inflammasome activation in SARS-CoV are also applicable in the case of SARS-CoV-2. Indeed, recent studies have shown that SARS-CoV-2 genome-encoded proteins, such as spike glycoprotein, can trigger the activation of NLRP3 inflammasome (Pan et al., 2020; Theobald et al., 2020).

In addition to the direct activation induced by genome-encoded proteins, SARS-CoV-2 can also activate NLRP3 inflammasome in an indirect way. For instance, it has been reported that MERS-CoV could activate NLRP3 inflammasome in a complement receptor-dependent manner (Jiang et al., 2019). The binding of SARS-CoV-2 spike glycoprotein with angiotensin-converting enzyme 2 (ACE2) triggers a series of complex molecular events, and ultimately leads to a hyper-inflammatory state. Both renin-angiotensin-aldosterone system (RAAS) and complement cascade are involved in SARS-CoV-2-induced overproduction of inflammatory cytokines (Mahmudpour et al., 2020), and this overproduction is caused by NLRP3 inflammasome (Ratajczak and Kucia, 2020). Besides, the ATP released by pyroptosis-related inflammatory cells also influences the activation of NLRP3 inflammasome via ATP-P2X7R pathway.

NLRP3 inflammasome plays a major role in mediating the immune response against SRAS-CoV-2. The inflammatory factors produced by NLRP3 inflammasome could promote antigen-presenting cells to upregulate co-stimulatory molecules (such as CD40, CD80 and CD86), thus strengthening the adaptive immune responses and enhancing viral clearance. In general, moderate inflammatory response is beneficial to eliminate the virus and repair the damaged tissue. However, if NLRP3 inflammasome has a low or impaired response to the virus, it will lead to a low-level of inflammatory response, which in turn weakens the immune system and protects the virus from clearance. For example, it has been reported that the leucine-rich repeat (LRR) domain mutation of bat NLRP3 inhibits the activation of NLRP3 inflammasome during MERS-CoV infection, leading to an impairment of the immune system (Ahn et al., 2019). Therefore, it is speculated that bats may carry MERS-CoV and other coronavirus without symptoms. On the other hand, overactivation of NLRP3 inflammasome produces excessive DAMPs, causing pyroptosis, neutrophil infiltration, macrophage activation, Th17 differentiation and excessive production of inflammatory cytokines, which ultimately leads to tissue damage and fibrosis (Lin et al., 2019; van den Berg and Te Velde, 2020). For example, IL-1β, which is induced by NLRP3 inflammasome activation to protect the host in the early phase of a viral infection, can also have serious negative consequences if the excessive production persists throughout the infection (Tate et al., 2016). Thus, it is necessary to control the activation of NLRP3 inflammasome.

Previous studies have reported that NLRP3 inflammasome and IL-1β are involved in lung injury and ARDS (Kolb et al., 2001; Ganter et al., 2008). The higher levels of Caspase-1 p20 and IL-18 indicated more severe symptoms and worse prognosis in patients with COVID-19 (Rodrigues et al., 2021), suggesting that NLRP3 inflammasome is not only activated but also plays a key role in COVID-19 progression. Moreover, dysregulation of NLRP3 inflammasome activity was observed in patients with severe COVID-19 (van den Berg and Te Velde, 2020). Hence, targeting NLRP3 inflammasome may be an effective strategy for the treatment of severe COVID-19 patients (Freeman and Swartz, 2020).

## Impact of Sex Differences on NLRP3 Inflammasome Activation Induced by SARS-CoV-2

**FIGURE 1:**
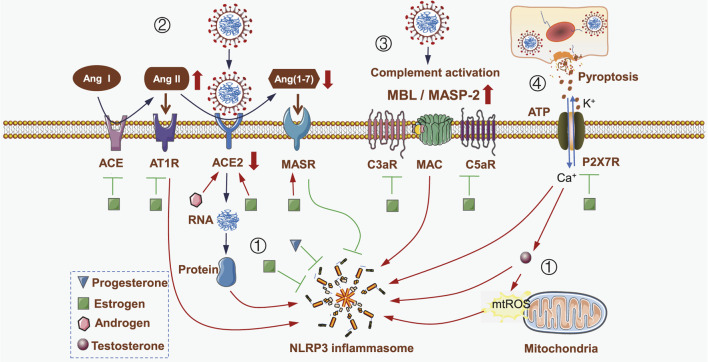
Impact of sex differences on NLRP3 inflammasome activities during SARS-CoV-2 infection. (i) SARS-CoV-2 genome-encoded proteins directly activate NLRP3 inflammasome. Estrogen and progestin directly inhibit NLRP3 inflammasome. However, the increased level of testosterone during SARS-CoV-2 infection promotes NLRP3 inflammasome activation in a ROS-dependent manner. (ii) SARS-CoV-2 hijacks ACE2 to mediate virus invasion and replication. ACE2 is then downregulated, leading to a decrease in Ang (1–7) level, and ultimately weakens MASR-mediated inhibition of NLRP3 inflammasome; meanwhile, Ang II is upregulated and interacts with AT1R to activate NLRP3 inflammasome. Both androgen and estrogen inhibit NLRP3 inflammasome by increasing ACE2 expression. However, estrogen also inhibits ACE and ATR1, while upregulates MASR, thus leading to NLRP3 inflammasome inhibition. (iii) SARS-CoV-2 infection induces the activation of complement cascade by increasing the levels of MBL, MASP-2, C3aR, C5aR and MAC, which in turn activates NLRP3 inflammasome. Estrogen inhibits the activation of complement cascade to inhibit NLRP3 inflammasome. (iv) SARS-CoV-2 infection induces pyroptosis and releases extracellular ATP, which acts on P2X7R and results in an ionic flux to activate NLRP3 inflammasome; meanwhile testosterone is induced to activate NLRP3 inflammasome. Estrogen inhibits P2X7R, thus suppressing the activation of NLRP3 inflammasome. Triangle represents progesterone, square represents estrogen, hexagon represents androgen, and circle represents testosterone*.*

Under disease conditions, NLRP3 inflammatory components are differently expressed in a sex-dependent manner and closely associated with the development of hyper-inflammation. For example, the expression levels of NLRP3, ASC, CASP1 and IL1B in male patients with abdominal aortic aneurysm were remarkably increased compared to those in female patients, and were closed related to disease progression (Wu et al., 2016). Moreover, the activity of NLRP3 inflammasome can be regulated by biological sex. A recent study demonstrated that male COVID-19 patients had higher plasma levels of innate immune cytokines, such as IL-8 and IL-18, along with more robust induction of non-classical monocytes (Takahashi et al., 2020), suggesting that sex difference is a major factor affecting the activation of NLRP3 inflammasome. Indeed, biological sex exerts a substantial impact on NLRP3 inflammasome activation. As shown in Figure 1, different NLRP3 inflammasome activating signals were respectively regulated by sex elements in varying degrees, thus leading to different degrees of inflammation and different types of symptoms in COVID-19 patients.

### Sex Differences Directly Affect NLRP3 Inflammasome Activation

Androgens are hormones that contribute to growth and reproduction in both men and women. Specifically, males tend to have higher androgen levels than females. Many studies have shown that androgen induces NLRP3 inflammasome activation in some disease conditions. For example, testosterone can directly activate NLRP3 inflammasome and mediate fibrosis in a CCl4-induced liver injury model (Ma et al., 2020). Excess testosterone production, which exceeds its normal range, can induce mitochondrial ROS to indirectly activate NLRP3 inflammasome (Alves et al., 2020). On the other hand, estrogen plays a more complicated role in regulating NLRP3 inflammasome activation. Specifically, estrogen induces NLRP3 inflammasome activation in tumor microenvironment (Wei et al., 2015; Liu et al., 2019; Wei et al., 2019), while inhibits this pathway under normal conditions. For instance, estrogen exhibits protective effects on brain by inhibiting NLRP3 inflammasome (Thakkar et al., 2016; Xu et al., 2016), and also demonstrates inhibitory effects at both mRNA and protein levels (Cheng et al., 2019). Besides, there are many factors that affect NLRP3 inflammasome activation (Zhang et al., 2020b), including ubiquitination and phosphorylation. The phosphokinases, such as JNK1, PKD and PKA, phosphorylate NLRP3 and promote its activation; while phosphatase, such as PP2A and PTPN22, mediate the dephosphorylation and suppression of NLRP3 (Song and Li, 2018). Estrogen downregulates receptor activator of nuclear factor-kappa B ligand (RANKL) to prevent RANKL-induced JNK activation (Srivastava et al., 2001), and its receptor directly binds to protein kinase D3 (PRKD3) gene promoter to decrease PKD3 expression (Borges et al., 2015), thus inhibiting phosphokinases-induced NLRP3 phosphorylation and activation. In addition, estrogen promotes the activity of PP2A (Ueda et al., 2013), thus leading to the dephosphorylation and impaired activation of NLRP3 inflammasome. Apart from estrogen, progesterone also inhibits NLRP3 inflammasome activation (Espinosa-Garcia et al., 2020). Notably, the levels of estrogen and progesterone decreased significantly after pausimenia (Harman et al., 2001; Galea et al., 2020), which in turn attenuated their protective effects. Furthermore, it has been reported that postmenopausal female patients with COVID-19 tend to share similar symptoms with male COVID-19 patients (Meng et al., 2020; Xie et al., 2020).

### Sex Differences Influence the Renin-Angiotensin-Aldosterone System to Activate NLRP3 Inflammasome

In addition to the above-mentioned direct effects, sex hormones also affect the activation of NLRP3 inflammasome via the RAAS, especially during SARS-CoV-2 infection. ACE2 can act as a receptor to mediate the invasion and replication of SARS-CoV-2 virus. In addition, ACE2 also plays a central role in maintaining the homeostasis of RAAS. This system begins with angiotensin, which is converted to angiotensin I by renin, angiotensin I is then converted to angiotensin II by ACE, and finally angiotensin II is converted to angiotensin (1–7) by ACE2. Angiotensin Ⅱ acts on AT1 and AT2 receptors, while angiotensin (1–7) acts on Mas receptor to neutralize angiotensin II/AT1R signal. The diminished level of ACE2 can break RAAS balance in angiotensin II/AT1R axis, while the increased level of ACE2 restores RAAS balance in angiotensin (1–7)/MASR axis to exert protective function (Patel et al., 2016).

In animal studies, ACE2 and AT2R have been shown to protect mice against ARDS caused by acid inhalation or sepsis, while other components of RAAS, such as ACE, angiotensin II and AT1R, can aggravate disease progression and lead to pulmonary edema and lung damage (Imai et al., 2005). The activation of ACE2/angiotensin (1–7)/MASR axis results in NLRP3 inflammasome inhibition, thus protecting from lipopolysaccharide-induced lung injury (Chen et al., 2019; Huang H. et al., 2020; Xu et al., 2020b; Zhao and Zhao, 2020). Furthermore, downregulation of ACE2 can lead to an imbalance of the RAAS and release of inflammatory cytokines (Mahmudpour et al., 2020).

During SARS virus infection, the level of ACE2 decreased significantly, while the level of ACE was not affected (Kuba et al., 2005), which made RAAS turn to angiotensin II/AT1R axis and elevated angiotensin II level can activate NLRP3 inflammasome (Zhang et al., 2016; Zhao et al., 2018; Wang et al., 2019), thus leading to development of ARDS. As similar to SARS-CoV, SARS-CoV-2 can also downregulate ACE2, and this downregulation is associated with disease severity (Kuba et al., 2005; Ahn et al., 2019; Zhang et al., 2020a). During SARS-CoV-2 infection, ACE2 interacts with SARS-CoV-2 spike protein to activate NLRP3 inflammasome, which in turn causes moderate inflammation (Ratajczak et al., 2020). A decrease in ACE2 inhibits the production of angiotensin (1–7), thereby exhibiting an inhibitory effect on NLRP3 inflammasome activation (Wen et al., 2016; Zhang et al., 2016; You et al., 2019).

Sex hormones regulate ACE2 expression to affect NLRP3 inflammasome activation. Both estrogen and androgen upregulate ACE2 expression (Bukowska et al., 2017; La Vignera et al., 2020). Male kidney ACE2 possesses higher activity than female (Liu et al., 2010), suggesting androgen possesses greater impact on ACE2 expression. However, RAS activation and angiotensin II upregulation are paralleled with renal injury in male, but not in female (Sullivan, 2008), suggesting that estrogen has a protective role in angiotensin II-induced kidney damage. Estrogen affects ACE-angiotensin II-AT1 axis by inhibiting ACE to reduce angiotensin II production (Bachmann et al., 1991; Brosnihan et al., 1999; Reckelhoff et al., 2000; Sullivan, 2008). In addition, estrogen decreases the expression of AT1R (Kisley et al., 1999; Wu et al., 2003) and increases the expression of ACE2, AT2R, MAS, angiotensin (1–7) and MASR (Cheng et al., 2015; Erfinanda et al., 2021). Hence, targeting SARS-CoV-2 infection-induced downregulation of ACE2 and angiotensin (1–7) as well as upregulation of angiotensin II can be an effective strategy to inhibit NLRP3 inflammasome activation.

### Sex Differences Regulate the Complement Cascade-induced NLRP3 Inflammasome Activation

During SARS-CoV-2 infection, some secondary factors also affect NLRP3 inflammasome activation. Specifically, NLRP3 inflammasome is required for the complement cascade-mediated induction of caspase-1 and IL-1β in intracerebral hemorrhage (Yao et al., 2017). The assembly of NLRP3 inflammasome requires the intracellular activation of C5 and stimulation of its receptor C5aR1 (Arbore et al., 2016), while C5aR2 promotes the expression of protein kinase R and contributes to NLRP3 inflammasome activation (Yu et al., 2019). Complement membrane attack complexes (MAC) assembles NLRP3 inflammasome and trigger IL-1 activation in IFN-γ-primed human endothelium (Xie et al., 2019). All these findings suggest that the complement cascade is involved in the activation of NLRP3 inflammasome.

NLRP3 inflammasome is activated in a complement-cascade dependent manner following MERS-CoV infection (Jiang et al., 2019). The increased levels of complement cascade components were found in COVID-19 patients, such as C5b-9, C4d and MASP2 (Magro et al., 2020), indicating that NLRP3 inflammasome may be activated in COVID-19 patients in a complement cascade-dependent manner.

Bacterial infection significantly upregulated the mRNA levels of complement cascade genes (C3-1, C3-3, Factor B and Factor H) in trout liver, while E2 treatment inhibited the upregulation of these genes (Wenger et al., 2011), suggesting that E2 could inhibit the activation of complement cascade. Moreover, there are significant sex differences in the abundance and function of complement cascade in healthy individuals. For example, lower levels of C3 and properdin were found in female subjects, which are responsible for alternative complement cascade activation (Gaya da Costa et al., 2018). In addition, the pro-inflammatory effect of C5b-9 is inhibited during disease in female subjects (Kotimaa et al., 2016), suggesting the involvement of sex differences in complement cascade activation. Given that the complement cascade is a source of sexual dimorphism in vulnerability to diverse illnesses (Kamitaki et al., 2020), it is speculated this system may play a key role in regulating sex differences among COVID-19 patients.

### Sex Differences Regulate ATP-P2X7R-Mediated NLRP3 Inflammasome Activation

During infection, ATP is released from the damaged cells to the extracellular environment. This extracellular ATP subsequently activates P2X7 receptor and generates an ionic flux that activates NLRP3 inflammasome. This effect is generally mild and difficult to observe. However, ionic flux is a common mechanism shared by other PAMPs and DAMPs, which have no structural similarity (Gong et al., 2018). This property made ATP-P2X7R signaling pathway synergistically interact with other NLRP3 inflammasome stimulus to initiate inflammatory responses. For example, gout arthritis is characterized by hyperuricemia-derived monosodium urate (MSU) deposition followed by NLRP3 inflammasome activation (Martinon et al., 2006), while MSU alone does not trigger gout flare. Further research showed that MSU synergizes with ATP to promote NLRP3 inflammasome activation and gout flare. Therefore, it is speculated that the function of P2X7R may determine whether hyperuricemia patients develop gout arthritis (Tao et al., 2013; Tao et al., 2017). In addition, there are differences in P2X7R functions among different individuals; thus, only hyperuricemia patients who have strong P2X7R function are prone to develop into gout. Based on this observation, COVID-19 patients who have strong P2X7R function may experience NLRP3 inflammasome overactivation and subsequently a “cytokine storm”, which represents a major mechanism underlying the disease exacerbation in some but not all male patients. Therefore, P2X7R can serve as a potential target for COVID-19 treatment.

During SARS-CoV-2 infection, testosterone level may be elevated because ACE-2 is expressed by Leydig cell and can be hijacked by the virus (Nashiry et al., 2021), which in turn leads to inflammatory pyroptosis in Leydig cells and increased extracellular ATP levels (Zhang et al., 2017). These extracellular ATPs activate P2X receptors expressed in Leydig cells (Antonio et al., 2009) and promote testosterone secretion (Foresta et al., 1996). Since testosterone exerts a promoting role in NLRP3 inflammasome activation, the elevated level of testosterone will further trigger NLRP3 inflammasome activation or even overactivation. In female subjects, estrogen impairs the function of P2X7R (Gorodeski, 2004), and inhibits the synergistic interaction between P2X7R and other NLRP3 inflammasome activating signals, thereby preventing NLRP3 inflammasome from overactivation. This may be an important reason why female COVID-19 patients are not likely to develop into severe disease.

In summary, sex hormones both directly and indirectly affect the activation of NLRP3 inflammasome through different mechanisms (Table 1). Besides, other mechanisms may also be involved. For example, heat shock protein 27 (HSP27) is a member of the small heat shock protein family that exerts extracellular anti-inflammatory effects (Batulan et al., 2016). A previous study has shown that HSP27 can alleviate SARS-CoV-2-induced cytokine storm by inhibiting NLRP3 inflammasome and other pathways (O'Brien and Sandhu, 2020). Since the extracellular release of HSP27 is largely dependent on estrogen levels (Rayner et al., 2008; Rayner et al., 2009), we speculate that estrogen can inhibit the activation of NLRP3 inflammasome by promoting HSP27 secretion.

**TABLE 1:** Regulatory effects of sex hormones on NLRP3 inflammasome activation.

	Influence way	Effect target	Activate/Inhibit	References
Estrogen	ATP-P2X7R-NLRP3	P2X7R	Inhibit	Gorodeski (2004)
	ACE-angiotensin II-AT1-NLRP3	ACE	Inhibit	Brosnihan et al. (1999), Kisley et al. (1999), Wu et al. (2003)
		AT1		
	ACE2-angiotensin (1–7)-NLRP3	ACE2	Inhibit	Bukowska et al. (2017)
	RANKL-JNK- NLRP3 phosphorylation	RANKL	Inhibit	Srivastava et al. (2001)
	PKD3-NLRP3 phosphorylation	PKD3	Inhibit	Borges et al. (2015)
	PP2A-NLRP3 dephosphorylation	PP2A	Inhibit	Ueda et al. (2013)
	HSP27-NLRP3	HSP27	Inhibit	Rayner et al. (2008), Rayner et al. (2009)
Progesterone	HMGB1-NLRP3	HMGB1	Inhibit	Espinosa-Garcia et al. (2020)
Testosterone	ROS-NLRP3	ROS	Activate	Chignalia et al. (2012)
	ATP-P2X7R-testosterone-NLRP3	Testosterone	Activate	Foresta et al. (1996), Antonio et al. (2009), Zhang et al. (2017)
	NLRP3	NLRP3	Activate	Ma et al. (2020)
	mROS-NLRP3	mROS	Activate	Alves et al. (2020)
Androgen	ACE2-angiotensin (1–7)-NLRP3	ACE2	Inhibit	La Vignera et al. (2020)

## Discussion

Since NLRP3 inflammasome activation is regulated by many factors, the above-mentioned mechanisms may not be able to explain all of the details. However, it is sufficient to say that the sex-related overactivation of NLRP3 inflammasome increases overall mortality in male COVID-19 patients, and NLRP3 inflammasome is activated during coronavirus infection. Compared with other coronavirus infections, the mortality of COVID-19 caused by SARS-CoV-2 is relatively lower, indicating a moderate activation of NLRP3 inflammasome in COVID-19 patients. Under this condition, sex influence is amplified that leads to different mortality between male and female COVID-19 patients, while SARS and MERS patients are not influenced by sex differences.

Since NLRP3 inflammasome is well controlled in most patients with COVID-19, cytokine storm is not common. In light with this, molecular factors that affect the overactivation of NLRP3 inflammasome are vital for predicting the outcome of COVID-19 patients. For example, in addition to biological sex, obesity-associated NLRP3 inflammasome activating factors also increase the risk of cytokine storms (López-Reyes et al., 2020). Moreover, older adults possess higher levels of NLRP3 inflammasome (Stout-Delgado et al., 2016; Lara et al., 2020) and are prone to develop cytokine storms, thus elderly patient with COVID-19 have worse prognosis. Hence, for male, elderly, obese and high-risk COVID-19 patients with NLRP3 inflammasome overactivation, drugs that target NLRP3 inflammasome may be used to prevent disease progression from mild to severe. Growing evidence has shown that colchicine inhibits NLRP3 inflammasome activation (Demidowich et al., 2016) and effectively reduces the overall mortality of COVID-19 patients (Reyes et al., 2020).

In addition, the use of hydroxychloroquine in COVID-19 treatment is still controversial, as it failed to prevent SARS-CoV-2 infection (Boulware et al., 2020). However, it do inhibit the activation of NLRP3 inflammasome (Tang et al., 2018). Considering the pivotal function of NLRP3 inflammasome in COVID-19, hydroxychloroquine could possibly inhibit NLRP3 inflammasome-induced cytokine storms and cause less severe symptoms (Lucchesi et al., 2020). Apart from colchicine and hydroxychloroquine, there are also other new or classic drugs that inhibit NLRP3 inflammasome and potentially effective to prevent the cytokine storms in COVID-19 patients (Zahid et al., 2019), including Tranilast and Oridonin. Furthermore, drugs like Anakinra that target the downstream effectors of NLRP3 inflammasome (such as IL-1β, IL-18 and their receptors) may also exert protective effects against SARS-CoV-2 infection (Jamilloux et al., 2020; Pontali et al., 2020; Soy et al., 2020).

Other than COVID-19, there are also NLRP3 inflammasome-associated diseases in which sex elements play a central role. For example, gout arthritis is characterized by MSU-stimulated NLRP3 inflammasome activation. This disease can also be influenced by biological sex, which is more common in men than in women. Sex elements affect gout flare by regulating NLRP3 inflammasome activation and ATP-P2X7R-NLRP3 pathway (Tao et al., 2013; Tao et al., 2017). Estrogen also inhibits the function of P2X7R (Gorodeski, 2004) to control gout flare, indicating the role of sex differences in this disease. However, inhibition of NLRP3 inflammasome in females is not always protective; in some cases, females are even more likely to develop infectious diseases. For example, Zika virus can activate NLRP3 inflammasome (Wang et al., 2018), and young women are more likely to be infected (Lozier et al., 2018). Given that the suppression of NLRP3 inflammasome by estrogen impairs the immune response against the virus and increases the risk of infection, this somehow explains that the majority of asymptomatic COVID-19 patients are females. Therefore, to avoid the damage caused by excessive inflammation, accurate detection of NLRP3 inflammasome is required to assess whether it exerts protective or detrimental role in SARS-CoV-2 infection. At the same time, appropriate interventions should be developed for the treatment of this disease.
